# Long term observation of MRSA prevalence in a German rehabilitation center: risk factors and variability of colonization rate

**DOI:** 10.3205/dgkh000281

**Published:** 2016-10-05

**Authors:** Jens Gieffers, André Ahuja, Ronald Giemulla

**Affiliations:** 1 Institute of Microbiology, Hygiene and Laboratory Medicine, Klinikum Lippe, Detmold, Germany; 2 Department of Life Science Technologies, Hochschule Ostwestfalen-Lippe, University of Applied Sciences, Lemgo, Germany; 3 Deutsches Beratungszentrum für Hygiene, Freiburg, Germany

**Keywords:** MRSA, prevalence, rehabilitation, Germany

## Abstract

**Background:** Data on MRSA prevalence in rehabilitation centers are sparse.

**Methods:** We screened more than 18,000 patients with neurological, cardiac/pulmonary or orthopedic diagnoses treated in three German rehabilitation centers and documented potential risk factors in almost 1,500 of them.

**Results:** 2.1% were MRSA positive (CI 1.9%–2.4%). Prevalence was higher in neurologic patients (3.7%) and lower in orthopedic patients (0.9%). While the overall MRSA situation was stable over two years, the weekly MRSA rate fluctuated strongly (0.0% to 8.0%).

We confirmed five risk factors in our study population. A risk adapted screening strategy derived from our data had a significance of 74% and a positive predictive value of only 2.2%.

**Conclusion:** MRSA positivity is a rare and highly variable event, requiring a huge sample size to generate robust data. The benefit of a risk-adapted screening strategy over a general screening should be questioned in each individual setting.

## Zusammenfassung

**Hintergrund:** Es liegen nur wenige repräsentative Daten zur MRSA-Prävalenz in Rehabilitationseinrichtungen vor.

**Methode:** Wir untersuchten mehr als 18.000 Patienten mit neurologischen, kardial/pulmonalen oder orthopädischen Diagnosen, die in drei deutschen Rehabilitationseinrichtungen behandelt wurden, und dokumentierten bei 1.500 Patienten mögliche Risikofaktoren.

**Ergebnisse:** 2,1% waren MRSA positiv (KI 1,9%–2,4%). Die Prävalenz war bei neurologischen Patienten höher (3,7%) und niedriger bei orthopädischen Patienten (0,9%). Während die Gesamt-MRSA-Prävalenz über den beobachteten Zweijahreszeitraum stabil war, schwankte die wöchentliche MRSA-Rate stark (0,0%–8,0%).

Wir identifizierten fünf Risikofaktoren in unserer Studienpopulation. Ein aus diesen Daten abgeleitetes risikoadaptiertes Screening hatte eine Signifikanz von 74%, aber einen positiven prädiktiven Wert von nur 2,2%.

**Fazit:** Da der MRSA-Nachweis selten und sehr variabel ist, ist ein großer Stichprobenumfang notwendig, um robuste Daten zu generieren. Der Vorteil eines risikoadaptierten Screenings gegenüber einem generellen Screening sollte in jeder individuellen Situation hinterfragt werden.

## Introduction

MRSA prevalence in hospitals is well documented. Data on rehabilitation centers are sparse though those patients might present a higher colonization rate due to their medical history. Moreover, many management strategies are based on data from a short observation period that are postulated to be representative. The aim of the present study was to assess risk factors of MRSA carriage on admission to rehabilitation centers and to determine the variability of the colonization rate over time.

## Methods

### Patient recruitment

We screened patients with neurological, cardiac/pulmonary or orthopedic diagnoses treated in three rehabilitation centers in the region of eastern Westphalia of Germany. The study was separated into two phases. In phase I, >95% of all admissions, a total of 1,464 patients, were screened and potential MRSA risk factors were documented. In phase II, >95% of all admissions of a period of two years, a total of 18,151 patients, were screened. 

#### Phase I

An MRSA screening including documentation of potential risk factors was conducted in the rehabilitation center A for 12 days in June 2012. A total of 186 patients with orthopedic (n=101) or cardiac/pulmonary indications (n=85) were examined. The study was repeated over a period of 13 weeks in autumn 2012 on 1,062 patients totally (477 patient with cardiac/pulmonary indications, 585 patients with orthopedic indications).

The following potential risk factors were documented: history of MRSA, chronic need for nursing care, hospitalization for more than 3 days within the last 12 months, antibiotic therapy within the last 6 months, chronic wounds, indwelling devices, contact with MRSA carrier, need for dialysis, contact with farm animals and absence of these risk factors.

Another MRSA screening including documentation of potential risk factors was conducted on 45 neurologic patients in the rehabilitation center B for 12 days in June 2012. 

In the rehabilitation center C, a total of 171 patients (82 patients with neurologic diagnoses, 89 patients with cardiac/pulmonary diagnoses) were screened and interviewed for potential risk factors for 24 days in February and March 2012.

Totally, 1,464 patients of three rehabilitation centers (651 patients (44.4%) with cardiac/pulmonary indications; 127 patients (8.7%) with neurologic indications and 686 patients (46.9%) with orthopedic indications) were assessed regarding MRSA status and potential risk factors.

#### Phase II

From January 2013 to December 2014 an MRSA screening was conducted on 18,151 patients of the above mentioned rehabilitation centers (representing >95% of all admissions). Risk factors were not assessed. 7,370 patients (40.6%) had a cardiac/pulmonary diagnosis, 5,949 (32.8%) an orthopedic and 4,832 (26.6%) a neurologic diagnosis.

### MRSA detection

Combined nasal/throat swabs were obtained on admission. A swap with clear Amies transport medium (UNI-TER) was used (MEUS s.r.l, Italy). Microbiological analysis was performed on CNA agar (Becton Dickinson GmbH, Heidelberg, Germany) and MRSA-Ident-Bouillon (heipha Dr. Müller GmbH, Eppelheim, Germany). MRSA was confirmed using Vitek 2 (Bio Mérieux, Nürtingen, Germany).

### Statistical analysis

Due to the rare appearance of MRSA, Fisher’s exact test (single sided) was applied to analyze risk factors. For each risk factor we separately estimated the critical significance for neglecting the hypothesis of independence. In according values of odds ratios allowed comparison of respective impacts. Further, the required sample size for a target accuracy of 0.5% for the 95% confidence interval for MRSA prevalence probability was estimated by use of beta distribution’s inverse. Accuracy here meant maximum absolute deviation from the respective point estimator given by the sample data.

## Results

### Phase I

Table 1 (Tab. 1) shows the potential risk factors of 1,464 screened patients and the rate of MRSA detection. 1.8% of all patients were MRSA positive. The rate was highest in neurological patients with 3.9% followed by patients with cardiac/pulmonary diagnoses with 2.5% and lowest in orthopedic patients with 0.9%. 

Five of the nine tested risk factors were associated with MRSA positivity (Table 2 (Tab. 2)). Correlation was strongest for “history of MRSA” followed by “chronic need for nursing care”, “hospitalization for more than 3 days within the last 12 months”, “antibiotic therapy within the last 6 months” and “chronic wounds”. In addition, correlation with “no risk factors” indicated, that more risk factors than the tested ones are present. No correlation with MRSA positivity was found for “indwelling devices”, contact with MRSA carrier”, “need for dialysis” and “contact with farm animals”.

All MRSA patients had one of the five confirmed risk factors. If “hospitalization for more than 3 days within the last 12 months” was excluded, as 82% of the admitted patients were positive for this factor, 20 out of 27 MRSA positive patients had a confirmed risk factor. Thus, the sensitivity of the screening strategy was 74%. On the other hand, risk factors were prevalent in MRSA negative patients, too. 62% (896 of 1,437) MRSA negative patients had one or more risk factors. The positive predictive value of the screening strategy was 2.2% only (data not shown).

### Phase II

Over a period of two years, all admissions were screened for MRSA. Of 18,151 patients 2.1% were positive with the 95%-confidence interval ranging from 1.9% to 2.4%. The rate was highest in neurological patients with 3.7% followed by patients with cardiac/pulmonary diagnoses with 2.1% and lowest in orthopedic patients with 0.9%. The weekly MRSA rate varied between 0.0 and 8.0% (range r=8.0%), but our statistical analysis showed that there was no decrease or increase of the weekly MRSA rate over the two year period (slope of the linear regression line d=–0.004; standard deviation 1.2%). We analyzed the set of data regarding the minimal sample size necessary to estimate the “true” prevalence of 2.1% on a confidence level of 95% and an absolute accuracy of ±0.5% (i.e. interval from 1.6% to 2.6%). In order to gain representative prevalence data, a minimum of 3,190 patients had to be screened in a population like ours. This required an average screening period of 18 weeks in our three rehabilitation centers. With an absolute accuracy of ±0.75% (i.e. interval from 1.35% to 2.85%), a minimum of 1,410 patients has to be screened lasting about 8 weeks in our setting. On the other hand, a one week observation period often required by quality management programs would deliver a confidence level of only 53% for a given accuracy of ±0.75% (interval of 1.5%). But such a low confidence level represents a state of low knowledge: The probability for the investigated parameter to be in the interval is almost the same as to be outside (data not shown).

## Discussion

In the present study, we investigated the MRSA prevalence in rehabilitation centers over a period of more than two years.

As one part of our study, we screened more than 18,000 patients and found 2.1% to be MRSA-positive. Due to the sample size, the 95%-confidence interval ranged from only 1.9% to 2.4%. Prevalence was higher in patients with neurologic disorders (3.7%), on average in patients with cardiac/pulmonary disorders (2.1%) and lower in orthopedic patients (0.9%). To our knowledge, this is the biggest study on MRSA prevalence in rehabilitation centers. Two German studies with 5,896 [1] and 6,985 patients [2] reported a prevalence of 1.2% (confidence interval not given) and 2.1% (confidence interval 0.3%–4.3%), respectively. Another German study [3] with 324 patients found a prevalence of 1.2% (confidence interval 0.4%–3.3%). In principle these results are not inconsistent to those of the present study as the respective sample origin has to be considered as well as the respective sample size, the latter one well known as an indicator for statistical credibility. The first study found in concordance to our one the highest prevalence in neurologic patients (4.1%) and a low prevalence of 1.2% in orthopedic patients. Cardiac patients had a prevalence of only 0.6% that was distinctly lower than in our study. 

The average MRSA rate in rehabilitation centers is comparable to that in German acute care hospitals that was found to range from 1.6% [4] to 2.2% [5]. In centers specialized on certain MRSA high risk groups like neurologic patients, prevalence might exceed the one of acute care hospitals. Prevalence in German rehabilitation centers seems to be distinctly lower than in other European countries (France 14.6%, Spain 8.1%, Italy 7.3% [6]). Noteworthy, these countries are reported to have a higher prevalence in acute care hospitals, too [7]. 

Another aim of the study was to assess the potential of a risk adapted screening strategy. The tested risk factors included in our questionnaire were derived from the German guideline [8]. We confirmed five of the nine potential risk factors in our study population. Interestingly, “contact with MRSA carrier” was no risk factor. When we reduced our data set to patients with the confirmed risk factors “history of MRSA”, “chronic need for nursing care”, “antibiotic therapy within the last 6 months” and “chronic wounds” the sensitivity of the screening strategy was 74% but the positive predictive value only 2.2%. In comparison to a general, not selected screening this strategy was able to reduce the tested patients to the half but missed one quarter of the MRSA patients. Thus, we conclude that in our setting a risk adapted screening is not reasonable. 

The variability of the weekly MRSA prevalence was given by a range of 8%, a mean of 2.1% but no trend over the two year study period. We calculated that the minimal sample size in order to determine the prevalence with a confidence level of 95% and ±0.5% accuracy would be almost 4,000 patients and would last in our setting 18 weeks of screening. This should be taken into consideration when risk-adapted screening strategies are derived from short periods of general screening.

The significance of studies like ours is influenced by the adherence to the study protocol. We have no data on how accurate the questionnaire on potential risk factors was completed and the nasal/throat swaps were obtained by different persons. The same swap material was used over the study period but we did not test the performance in comparison to other products. Moreover, the low prevalence of MRSA is a problem affecting all studies like this. We dealt with it by determining the prevalence in the huge study population overcoming the high variability and resulting in a narrow confidence interval. But interviewing the same number of patients was not feasible, thus, risk factors were determined only in a low number of individuals.

## Conclusions

We reported a MRSA prevalence in rehabilitation centers of 2.1% on the basis of more than 18,000 patients. Prevalence was higher in neurologic and lower in orthopedic patients. While the overall MRSA situation was stable, the weekly MRSA rate fluctuated strongly, thus, a sufficient sample size is crucial for reliable results. We confirmed five risk factors in our study population. Nevertheless, a risk adapted screening strategy derived from our data had a significance of 74% and a positive predictive value of only 2.2%. Thus, the benefit of a risk adapted screening over a general screening must be questioned.

## Notes

### Competing interests

The authors declare that they have no competing interests.

## Figures and Tables

**Table 1:**
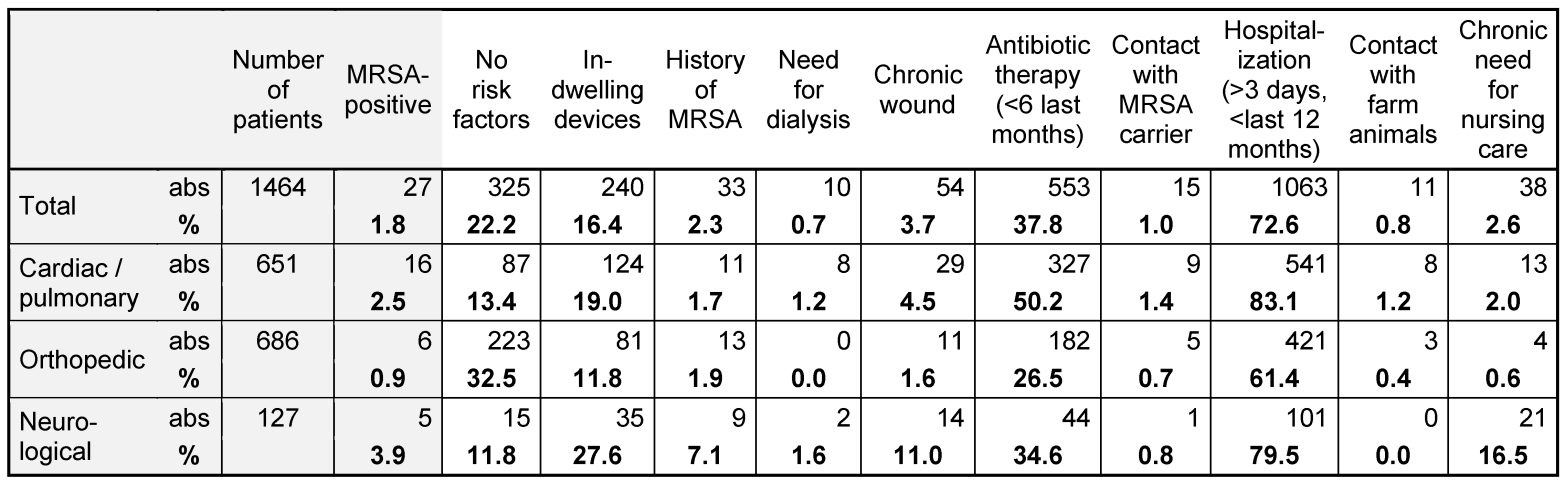
MRSA prevalence and postulated risk factors

**Table 2:**
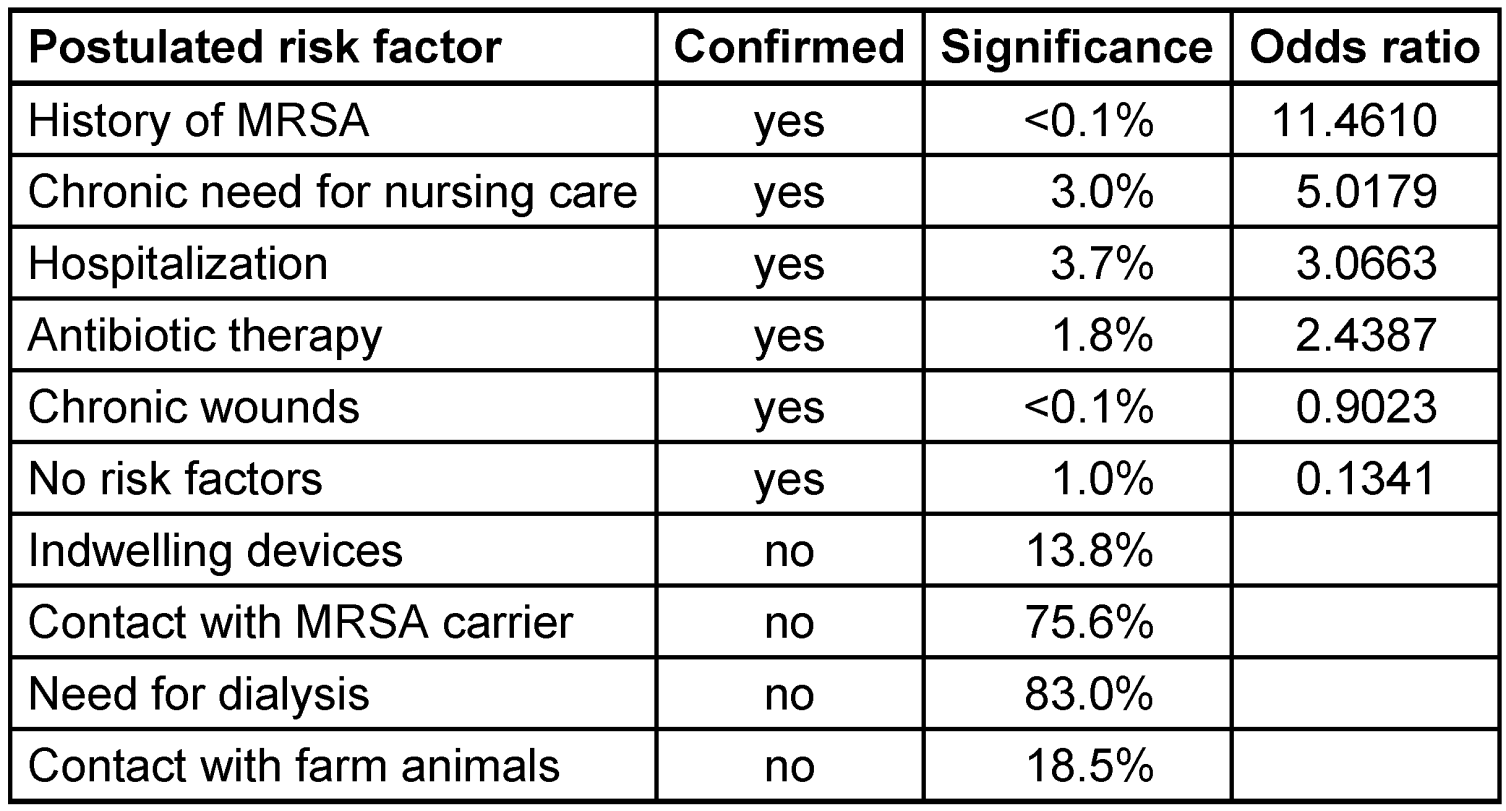
Statistical analysis of risk factors for MRSA positivity
